# Regulation of 11β-Hydroxysteroid Dehydrogenase Type 1 and 7α-Hydroxylase CYP7B1 during Social Stress

**DOI:** 10.1371/journal.pone.0089421

**Published:** 2014-02-21

**Authors:** Martin Vodička, Peter Ergang, Anna Mikulecká, Lenka Řeháková, Petra Klusoňová, Jakub Makal, Matúš Soták, Jana Musílková, Petr Zach, Jiří Pácha

**Affiliations:** 1 Institute of Physiology, Academy of Sciences of the Czech Republic, Prague, Czech Republic; 2 Department of Physiology, Faculty of Science, Charles University, Prague, Czech Republic; 3 Institute of Anatomy, Third Faculty of Medicine, Charles University, Prague, Czech Republic; Max Planck Institute of Psychiatry, Germany

## Abstract

11β-hydroxysteroid dehydrogenase type 1 (11HSD1) is an enzyme that amplifies intracellular glucocorticoid concentration by the conversion of inert glucocorticoids to active forms and is involved in the interconversion of 7-oxo- and 7-hydroxy-steroids, which can interfere with the activation of glucocorticoids. The presence of 11HSD1 in the structures of the hypothalamic-pituitary-adrenal (HPA) axis suggests that this enzyme might play a role in the regulation of HPA output. Here we show that the exposure of Fisher 344 rats to mild social stress based on the resident-intruder paradigm increased the expression of 11HSD1 and CYP7B1, an enzyme that catalyzes 7-hydroxylation of steroids. We found that social behavioral profile of intruders was significantly decreased whereas their plasma levels of corticosterone were increased more than in residents. The stress did not modulate 11HSD1 in the HPA axis (paraventricular nucleus, pituitary, adrenal cortex) but selectively upregulated 11HSD1 in some regions of the hippocampus, amygdala and prelimbic cortex. In contrast, CYP7B1 was upregulated not only in the hippocampus and amygdala but also in paraventricular nucleus and pituitary. Furthermore, the stress downregulated 11HSD1 in the thymus and upregulated it in the spleen and mesenteric lymphatic nodes whereas CYP7B1 was upregulated in all of these lymphoid organs. The response of 11HSD1 to stress was more obvious in intruders than in residents and the response of CYP7B1 to stress predominated in residents. We conclude that social stress induces changes in enzymes of local metabolism of glucocorticoids in lymphoid organs and in brain structures associated with the regulation of the HPA axis. In addition, the presented data clearly suggest a role of 11HSD1 in modulation of glucocorticoid feedback of the HPA axis during stress.

## Introduction

A large number of studies have shown that different stressors produce profound physiological and behavioral disturbances that may contribute to psychopathology [1], [2] and alterations in immune system functionality [3]. Stress activates the sympatho-adrenomedullar and the hypothalamic-pituitary-adrenocortical (HPA) systems. The HPA axis is self-regulatory, utilizing its end products, cortisol and corticosterone, to control its own activation and responsiveness through a negative feedback mechanism. The neurons of the paraventricular nucleus represent the central coordinator of the HPA axis that is not only driven by negative corticosteroid feedback signals, but also by central stress excitatory and inhibitory circuits that are activated by stressors in both intrahypothalamic (arcuate nucleus, dorsomedial hypothalamus) and extrahypothalamic structures, in particular, the limbic structures (medial prefrontal cortex, hippocampus, amygdala) [4], [5]. These structures express glucocorticoid receptors that contribute to feedback control of the HPA axis [6].

The response of the target cells to the glucocorticoid signal does not only depend on the plasma level of the hormone and the density of corticosteroid receptors, but also on the intracellular concentration of glucocorticoids, which is predominantly determined by the local pre-receptor steroid metabolism. This metabolism depends on 11β-hydroxysteroid dehydrogenase (11HSD), an enzyme that exists in two forms - type 1 (11HSD1) and type 2 (11HSD2). 11HSD1 generally catalyses the reduction of biologically inactive 11-oxo-steroids cortisone and 11-dehydrocorticosterone to cortisol and corticosterone and thus increases the local concentration of active corticosteroids. In contrast, 11HSD2 operates as a strict dehydrogenase that oxidizes corticosterone and cortisol to 11-oxo-derivatives [7]. 11HSD1 is expressed in the brain [8]–[10], pituitary [11], [12], adrenal gland [13], and many peripheral organs including the spleen, thymus, and lymphatic nodes [14], [15].

The presence of 11HSD1 in the structures of the HPA axis suggests that the enzyme might play a role in the regulation of HPA output. Indeed, 11HSD1 knock-out mice exhibit attenuated glucocorticoid feedback on the HPA axis and exaggerated glucocorticoid response to stress [16], however, the genetic background of the mice significantly modulates their response to 11HSD1 deletion [17]. These findings indicate a potential role of 11HSD1 in stress-induced alterations of the HPA axis. However, previous studies investigating the effect of stress and glucocorticoids on 11HSD1 have been incomplete and contradictory [18]–[21]. They differed in the type and duration of stress applied, animal species and type of cells in which 11HSD1 was assessed. Until now, only one study has addressed the topic of chronic social stress on 11HSD1 in the hippocampus [20], even though social stressors have a profound influence on behavior, immunity, and physiology [22], [23].

As the most common stressors for humans are psychosocial in nature, we used a model of social stress based on a resident-intruder paradigm with the aim of evaluating the consequences of repeated mild/moderate stress on the regulation of 11HSD1 expression in brain structures associated with the HPA axis and in the adrenal glands. As social stress has been shown to have a profound influence on immune and inflammation responses [24], [25], the effect of social stress on 11HSD1 was also assessed in primary and secondary lymphoid organs. In addition, we investigated the effect of repeated social stress on the expression of CYP7B1, which catalyzes the 7α-hydroxylation of C_19_ and C_21_ steroids, and is expressed in various tissues including the brain. These 7-hydroxy-steroids interact with 11HSD1 and their presence can interfere with the activation of 11-hydroxy-steroids from 11-oxo-steroids catalyzed by 11HSD1 [26].

## Materials and Methods

### Animals

The experiments were performed on 65-day-old male (n = 21) Fisher 344 rats (Charles River, Germany). Animals were housed in groups of three to four in a temperature-controlled room on a 12/12-h light/dark cycle with *ad libitum* access to food and water throughout the entire study. They were left for three weeks to acclimatize before any experimental procedures. The animals were randomly assigned to one of three groups: (1) controls (2) residents, and (3) intruders, each consisting of seven animals. The protocol of the experiments was approved by the animal Care and Use Committee of the Institute of Physiology to be in agreement with the Animal Protection Law of the Czech Republic, which is fully compatible with the guidelines of European Community Council Directive 86/609/EEC. All efforts were made to minimize the animal suffering and to reduce the number of animal used.

### Resident-intruder Paradigm

The general design of the test was adapted from [27]. Briefly, if an unfamiliar conspecific intruder is introduced into the home cage of an isolated resident, intense social behavior arises. Such behavior is mainly initiated by the resident animal, indicating territorial advantage. This territorial advantage is obvious after a few days of the resident being isolated. The test relies on the concept of the ethological analysis of rodent behavior and can be used as a model of “social anxiety”. At the beginning of the experiment the resident rats were housed individually for one week, the intruders were housed in groups of three or four. Following the seven-day isolation period of the residents, the social encounter was performed for seven consecutive days, and arranged to ensure that each intruder rat met each of the corresponding residents for 30 minutes. The resident rats remained isolated in their home cages throughout the experiment, while the intruders were returned to their respective groups. There was no difference in the body weight of the residents (222.1±1.0 g) and intruders (218.5±1.5 g) after exposure to social stress and the weights were similar to control unstressed animals of the same age.

### Behavioral Testing

To compare social behavior between the first and the final social interaction (Session 1 vs. Session 7) the behavior was video-recorded for 15 minutes and the test was performed under low light intensity (35–45 lx) between 9.00 and 12.00 AM. The social behavioral patterns displayed were subsequently analyzed in detail by two trained experimenters using the computerized behavioral analysis system Observer (Noldus Information Technology, Wageningen, The Netherlands). Behavior was scored separately for each member of a pair (resident and intruder) except for wrestling, as this pattern involved two animals concurrently performing the same behavior. The number and the duration of the following exhibited patterns were evaluated: following/chasing (the pursuit of one rat by another) grabbing (rat grabbing the fur of another in the region of the neck), wrestling (both animals roll and tumble with one another), on-top posture (one rat positioned over another with forepaws placed on it), and digging (moving substrate forward with front paws and nose, or backwards with hind paws), a non-social behavior observed only in intruders.

### Brain Sampling and Processing

Intact animals (controls) and the rats after recording the last social interaction session (Session 7) were immediately anesthetized with isoflurane and blood was collected by cardiac puncture. Then the rats were killed by decapitation and the brain and selected other tissues were removed and immediately frozen in liquid nitrogen.

**Figure 1:**
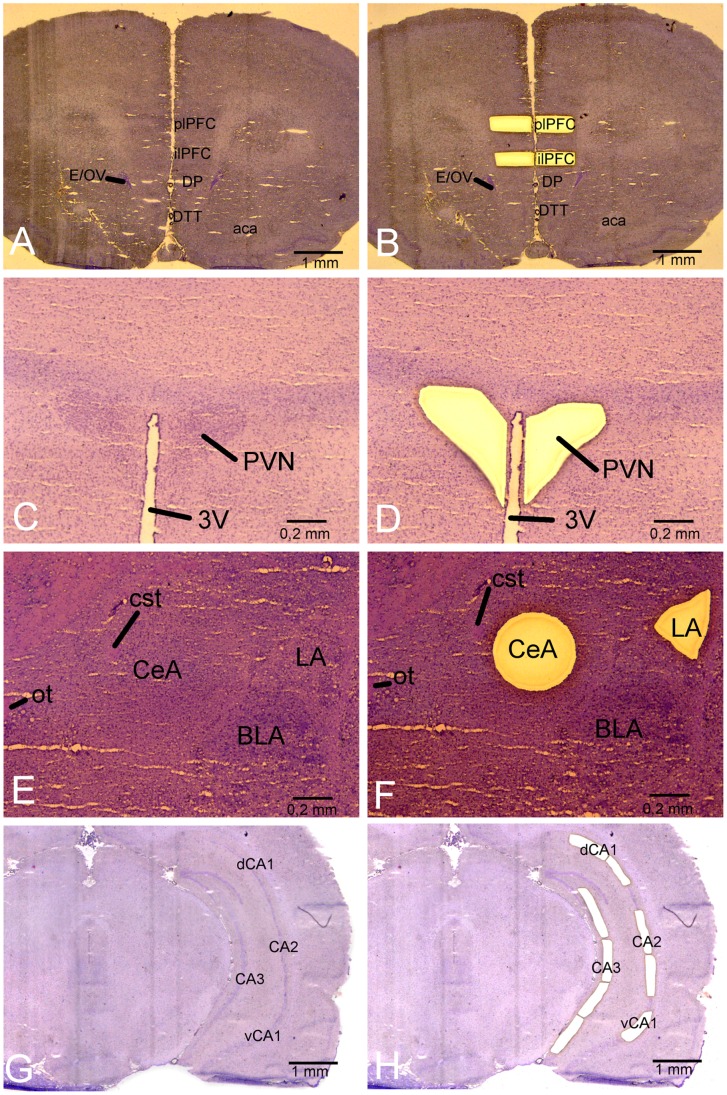
Laser capture microdissection of cell populations from the prelimbic and infralimbic cortex (A, B), hypothalamic paraventricular nucleus (C, D), central and lateral nucleus of amygdala (E, F) and CA1, CA2 and CA3 regions of hippocampus (G, H). Representative 20-µm-thick coronal sections of unfixed, frozen rat brain stained with cresyl violet are shown before (left panel) and after capturing (right panel). The dissected regions were captured into the microcentrifuge tubes and used for total RNA isolation and RT-PCR analysis. *Abbreviations*: aca, anterior commissure; BLA, basolateral amygdala; CA2 and CA3, hippocampal CA2 and CA3 regions; CeA, central nucleus of amygdala; cst, commissural stria terminalis; dCA1, dorsal part of CA1 hippocampus; DP, dorsal peduncular cortex; DTT, dorsal tenia tecta; E/OV, ependymal and subependymal layer of olfactory ventricle; ilPFC, infralimbic prefrontal cortex; LA, lateral nucleus of amygdala; ot, optic tract; plPFC, prelimbic prefrontal cortex; PVN, paraventricular hypothalamic nucleus; vCA1, ventral part of CA1 hippocampus; 3V, 3rd ventricle;

Brain specimens of hypothalamic paraventricular nucleus (PVN), central (CeA) and lateral amygdala (LA), prelimbic (plPFC) and infralimbic prefrontal cortex (ilPFC), hippocampal CA2 and CA3 regions, and ventral (vCA1) and dorsal (dCA1) parts of CA1 region, were prepared by laser microdissection and RNA analysis was performed as previously described, with some modifications [28]. Briefly, coronal brain sections (20 µm) were serially cut with a cryostat at −19°C. The regions were identified based on standard anatomical landmarks and stereotaxic coordinates (see Table 1, Fig. 1) according to Paxinos and Watson [29]. The sections of the studied structures were mounted onto slides coated with polyethylene naphthalate membrane (Leica Microsystems, Wetzlar, Germany), fixed in 95% ethanol, stained with 4% cresyl violet acetate and washed three times in 95% ethanol. The studied brain structures were dissected using a LMD6000 Laser Microdissection System (Leica) and captured into the caps of the microcentrifuge tubes. Microdissected tissues were homogenized in 75 µl RLT buffer (Qiagen, Hilden, Germany) and stored at −80°C until RNA isolation.

**Table 1:** Rat brain regional coordinates.

Region	Bregma coordinates	ML	DV	Figure no.
plPFC	3.00 to 2.50 mm	±(0 to 1.0)	3.2 to 4.5	10–12
ilPFC	3.00 to 2.50 mm	±(0 to 0.9)	4.5 to 5.2	10–12
CeA	−2.56 to −2.76 mm	±(3.8 to 4.8)	7.5 to 8.6	54–56
LA	−2.56 to −2.76 mm	±(5.2 to 5.8)	7.4 to 8.4	54–56
dCA1	−4.92 to −4.97 mm	±(3.5 to 5.5)	2.8 to 4.0	74
vCA1	−4.92 to −4.97 mm	±(4.8 to 6.2)	6.8 to 8.8	74
CA2	4.92 to 4.97 mm	±(5.3 to 5.9)	5.3 to 5.9	74
CA3	4.92 to 4.97 mm	±(3.5 to 5.0)	5.0 to 7.0	74
PVN	−1.72 to −1.80 mm	±(0 to 0.5)	7.8 to 8.6	47–48

plPFC, prelimbic prefrontal cortex; ilPFC, infralimbic prefrontal cortex; CeA, central amygdala; LA, lateral amygdala; dCA1 and vCA1, dorsal and ventral parts of hippocampal CA1 region; CA2, hippocampal CA2 region; CA3, hippocampal CA3 region; PVN, paraventricular nucleus. The extent of the dissected regions is characterized according to the mediolateral (ML) and dorsoventral (DV) axis. Coordinates and figure numbers are based on the atlas “The Rat Brain In Stereotaxic Coordinates. 6^th^ Edition by G. Paxinos & C. Watson. Elsevier, 2007”. The thickness of the brain coronal sections was 20 µm and the total area of the isolated brain structure was 0.12 to 0.60 mm^2^.

Total RNA was isolated from the captured tissue using an RNeasy Micro Kit (Qiagen, Hilden, Germany) and evaluated with a NanoDrop spectrophotometer (NanoDrop Products, Wilmington, DE, USA). The RNA samples were reverse-transcribed to cDNA with Enhanced Avian Reverse Transcriptase (Sigma-Aldrich, St. Louis, MO, USA). Because the RNA yield of cytokine transcripts was low, an aliquot of the cDNA sample was amplified with TaqMan PreAmp Master Mix Kit (Life Technologies, Carlsbad, CA, USA) according to the manufacturer’s instructions. The cDNA samples were analyzed by real-time PCR on an ABI PRISM 7000 Sequence Detection System (Applied Biosystems, Foster City, CA, USA) using TaqMan Gene Expression Master Mix and TaqMan Assays (Life Technologies) specific for rat 11HSD1 (cat.no. Rn01461862_m1), 7-hydroxylase (CYP7B1; cat.no. Rn00567167_m1), glucocorticoid receptor (GR; cat.no. Rn00561369_m1), interleukin 1β (IL-1β; cat.no. Rn01514151_m1), tumor necrosis factor α (TNFα; cat.no. Rn 99999017_m1), osteopontin (OPN; cat.no. Rn01449972_m1), corticotropin-releasing hormone (CRH; cat. no. Rn01462137_m1) and CRH receptor 1 (CRHR1; cat. no. Rn00578611_m1). The housekeeping gene, glyceraldehyde-3-phosphate dehydrogenase (GAPDH; TaqMan Endogenous Control cat. No. 4352338), was measured to normalize the mRNA expression in each sample, as its transcript is not changed in the rat brain during stress [30]. A single PCR reaction was performed in a final volume of 30 µl using target gene probes labeled with FAM in duplex with a GAPDH probe (VIC/MGB). The quantity of the transcript was determined using the standard curve method with 10-fold dilutions of the mixed cDNA sample.

### Peripheral Tissues Collection and Processing

Tissue samples of the anterior pituitary, adrenal gland, thymus, spleen and mesenteric lymphatic nodes (MLN) were snap-frozen and stored in liquid nitrogen. To separate the adrenal cortex and medulla, the samples of the adrenal gland were laser-microdissected and the samples of cortex and medulla were processed identically to the brain samples mentioned above. RNA from the pituitary and lymphoid organs was isolated using a GeneElute Mammalian Total RNA Miniprep Kit (Sigma Aldrich) and 0.1 µg of pituitary RNA and 1.0 µg of spleen, thymus and MLN RNA were reverse-transcribed using a High Capacity cDNA Reverse Transcription Kit and random hexamers (both Life Technologies). The mRNA levels were quantified by real-time PCR with TaqMan Gene Expression Master Mix and the TaqMan Assays mentioned above in a LightCycler 480 (Roche, Mannheim, Germany) or ABI PRISM 7000 Sequence Detection System (Applied Biosystems). The reactions were performed in 30 µl aliquots on a 96-well optical reaction plate containing TaqMan Gene Expression Master Mix with AmpErase UNG (Applied Biosystems), cDNA and TaqMan probes as mentioned above. The standard curve method was used to analyze the relative gene expression and the genes of interest were normalized to GAPDH.

### Plasma Corticosterone Measurement

Blood was collected in tubes containing K_2_EDTA and centrifuged for 10 min at 3,000 g. Plasma was aliquoted and kept frozen at −80°C until the assay. Corticosterone was measured using a commercial RIA kit (MP Biomedicals, Solon, OH, USA).

### Statistical Analysis

Data are presented as means ± SEM. The behavioral variables were analyzed with a two-way repeated measure ANOVA followed by Student-Newman-Keuls post-hoc test, where the type of condition (resident vs. intruder) was the between-subject factor and the time of measurement (session) was the within-subject factor. Digging behavior was analyzed by a one-way ANOVA because this behavior was observed only in intruder rats. Similarly, a one-way ANOVA and a *post hoc* multiple comparison test (Student-Newman-Keuls) were used for studies involving single comparisons of mRNA levels and plasma levels of corticosterone. All analyses were carried out using the software Statistica 6.1. (StatSoft Inc., Tulsa, OK, USA). The significance level was set at P<0.05.

## Results

### Effect of Repeated Social Stress on Behavior

**Figure 2:**
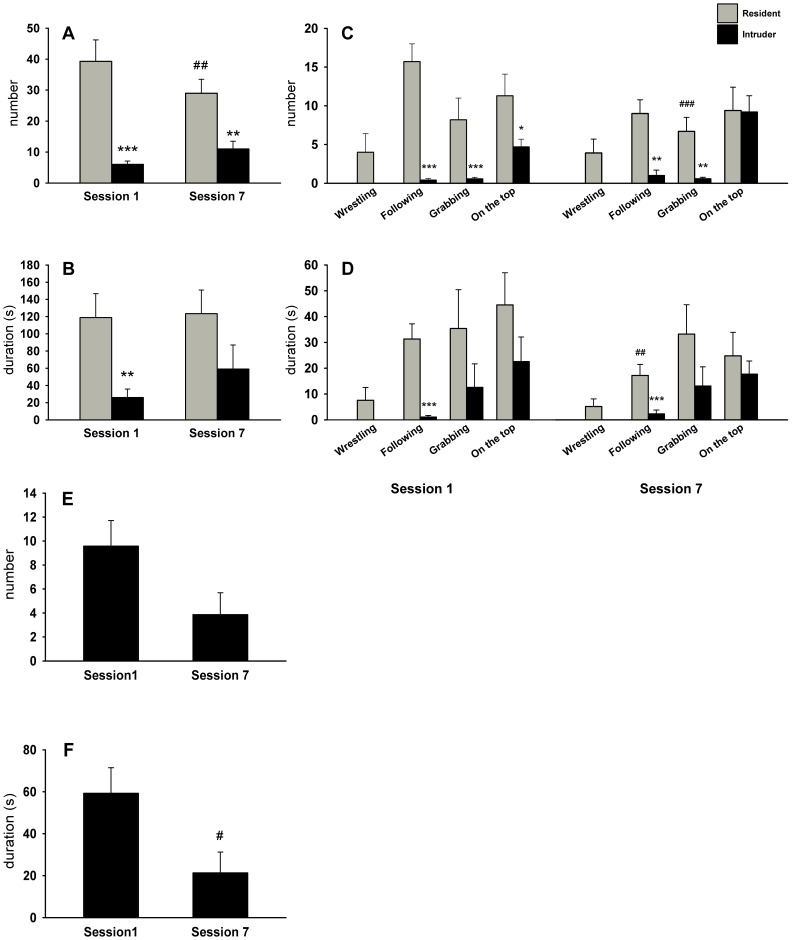
Behavioral differences in Fisher 344 rats on the resident-intruder test. Left side up: Total number (**A**) and total duration (**B**) of all social behavioral patterns displayed by resident and intruder rats. Right side up: Total number (**C**) and total duration (**D**) of individual behavioral patterns exhibited during social interaction. Left side down: Total number (**E**) and total duration (**F**) of digging patterns displayed only by intruder rats. Session 1: the first day, session 7: the last day. The values are expressed as means ± SEM. Significant differences between residents and intruders: *P<0.05, **P<0.01, ***P<0.001, and between Session 1 and Session 7: ^#^P<0.05, ^##^P<0.01, ^###^P<0.001.

The differences in the behavioral profile of resident and intruder rats are illustrated in Fig. 2. To simplify the text, only statistically significant differences were stated. ANOVA showed a significant effect on social interaction in the number as well as in the duration of all evaluated social behavioral patterns (*F*_1,12_ = 29.52, *P*<0.001; *F*_1,12_ = 10.18, *P*<0.01, respectively). In the first social session, the *post hoc* test revealed a significant decrease in both the number and the duration of all social behaviors displayed by intruders compared to residents (Fig. 2A,B). In the final encounter (Session 7; Fig. 2A,B), the intruder rats had a significant decrease in the number but not in the duration of social behavior. Further, the number of all behavioral patterns displayed by residents in the final seventh session was lower that in the first session.

The analysis of individual behavioral patterns revealed the condition (resident vs. intruder) to have a major effect on the number and the duration of following (*F*_1,12_ = 37.14, *P*<0.001; *F*_1,12_ = 26.91, *P*<0.001, respectively) and on the number as well as the duration of grabbing (*F*_1,12_ = 7.99, *P*<0.01; *F*_1,12_ = 5.03, *P*<0.05, respectively). The overall analysis did not reveal any significant differences in either the number or the duration of the on-top posture. In intruders, the *post hoc* test showed a decrease in the number of following and grabbing behaviors but only in the duration of following in both sessions. As for wrestling, no difference was detected between Session 1 and Session 7. Finally, digging (Fig. 2E, F), an index of anxiety-like behavior, was observed only in intruder rats and its duration was significantly decreased in Session 7 compared to Session 1 (*F*_1,6_ = 8.49, P<0.05).

### Effect of Repeated Social Stress on Plasma Corticosterone and Expression of 11HSD1, GR and Cytokines

**Figure 3:**
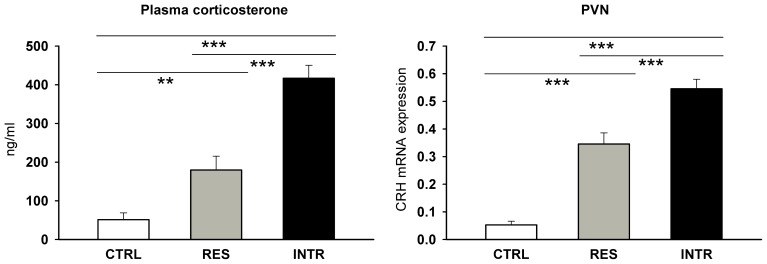
Plasma level of corticosterone and expression of corticotropin-releasing hormone (CRH) in hypothalamic paraventricular nucleus of control (CTRL), resident (RES) and intruder rats (INTR) after the last social session. All values are means ± SEM. **P<0.01, ***P<0.001.

The repeatedly stressed rats had significantly elevated plasma corticosterone compared to intact control animals and the plasma levels in intruders were significantly higher than in residents (Fig. 3; F_2,18_ = 40.03, P<0.0001). Similarly, stress significantly increased the expression of CRH in PVN and the levels were significantly higher in intruders than in residents (Fig. 3; F_2,13_ = 52.53, P<0.0001). As social status plays an important role in determining the impact of stress on brain cytokines [31], [32], the expression of proinflammatory cytokines IL-1β, TNFα and OPN was measured in the PVN and CA1 region of the hippocampus. In intact controls, the expression of cytokines was absent or very low in both the PVN and CA1. Social interactions were followed by the upregulation of TNFα, IL-1β and OPN, and the effect did not differ between resident and intruder rats (Table 2). For comparative reasons we measured also expression of IL-1β in MLN (Table 2) and found out that stress did not upregulate cytokine expression in this tissue.

**Table 2:** Expression of pro-inflammatory cytokines tumor necrosis factor α (TNFα), interleukin 1β (IL-1β) and osteopontin (OPN) in paraventricular nucleus (PVN), CA1 region of ventral hippocampus (vCA1) and in mesenteric lymphatic nodes (MLN).

	PVN		vCA1		MLN	
	CTRL	Experimental	CTRL	Experimental	CTRL	Experimental
TNFα	n.f.	0.25±0.12	n.f.	0.38±0.14	n.m.	n.m.
IL-1β	0.02±0.01	0.23±0.10*	n.f.	n.f.	5.03±0.42	4.51±0.35
OPN	0.03±0.02	5.83±1.84**	0.01±0.00	2.03±0.65**	n.m.	n.m.

Data are means ± SEM (CTRL, n = 5–7; Experimental = 12–14); as upregulation of the transcripts was not significantly different in residents and intruders, both groups were merged; n.f., no signal of the transcript was found either in standard or in preamplified samples; n.m., the transcript was not measured in the samples. Quantitative PCR was measured in standard (MLN) or preamplified samples (PVN, vCA1) of intact controls and experimental animals (residents, intruders) as mentioned in Materials and Methods. *P<0.05, **P<0.01.

**Figure 4:**
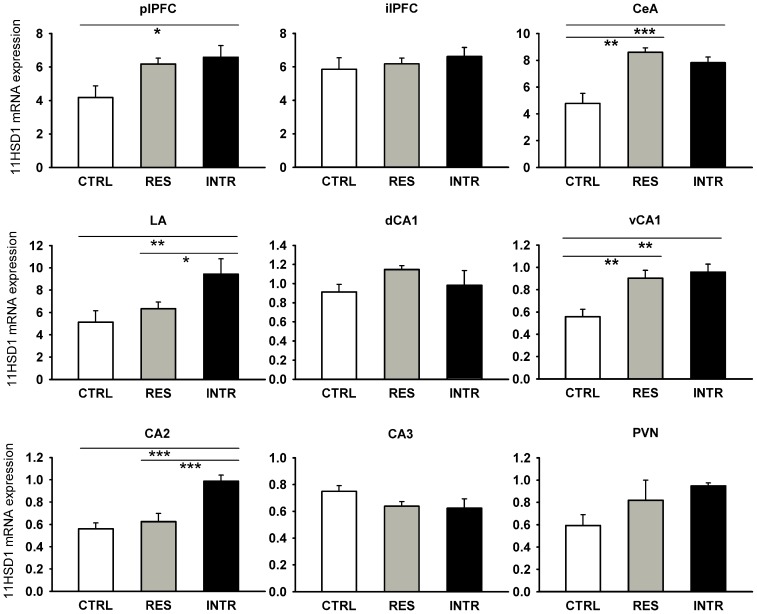
Effect of repeated social stress on expression of 11HSD1 in brain structures associated with the HPA axis. CTRL, control rats; RES, resident rats; INTR, intruder rats; plPFC, prelimbic prefrontal cortex; ilPFC, infralimbic prefrontal cortex; CeA, central amygdala; LA, lateral amygdala; dCA1 and vCA1, dorsal and ventral parts of CA1 hippocampus; CA2 and CA3, hippocampal CA2 and CA3 regions; PVN, paraventricular nucleus. All values are means ± SEM. *P<0.05, **P<0.01, ***P<0.001.

Given that stress activates HPA axis that is self-regulated through negative feedback mechanism utilizing its end products, cortisol and corticosterone, we hypothesized that stress might modulate the feedback signaling in the HPA axis. To test this hypothesis, we analyzed the effect of stress on the expression of 11HSD1 and GR in PVN, prefrontal cortex, amygdala and hippocampus. As shown in Fig. 4, the expression of 11HSD1 was affected by social stress in some brain structures associated with regulation of the HPA axis. In particular, the 11HSD1 transcript was upregulated in the CeA and LA (F_2,12_ = 11.57; P<0.01 and F_2,17_ = 4.80; P<0.05, respectively), in the plPFC (F_2,14_ = 3.95; P<0.05), in the vCA1 (F_2,15_ = 8.38; P<0.001), but not dCA1 subfield of hippocampus, and in the CA2 hippocampus (F_2,15_ = 12.67; P<0.0001). Stress did not change the expression of 11HSD1 in the CA3 hippocampal region and PVN, even if the effect in PVN was just shy of statistical significance. Similarly, expression of GR (Table 3) was not changed by social stress in any investigated brain area with the exception of the vCA1 region of the hippocampus (F_2,18_ = 5.20; P<0.01).

**Table 3:** A comparison of glucocorticoid receptor mRNA expression in brain structures associated with the HPA axis in residents, intruders and unstressed control rats.

	Residents	Intruders	Controls
plPFC	0.15±0.01	0.16±0.02	0.13±0.03
ilPFC	0.17±0.01	0.16±0.01	0.17±0.02
CeA	0.31±0.06	0.34±0.01	0.26±0.03
LA	0.11±0.02	0.10±0.01	0.11±0.02
dCA1	0.70±0.07	0.92±0.07	0.78±0.07
vCA1	0.49±0.06**	0.41±0.04**	0.26±0.04
CA2	0.23±0.03	0.26±0.06	0.25±0.04
CA3	0.19±0.01	0.17±0.03	0.17±0.02
PVN	0.31±0.02	0.25±0.01	0.22±0.05

plPFC, prelimbic prefrontal cortex; ilPFC, infralimbic prefrontal cortex; CeA, central amygdala; LA, lateral amygdala; dCA1 and vCA1, dorsal and ventral parts of hippocampal CA1 region; CA2, hippocampal CA2 region; CA3, hippocampal CA3 region; PVN, paraventricular nucleus. All values are means ± SEM. **P<0.01.

**Figure 5:**
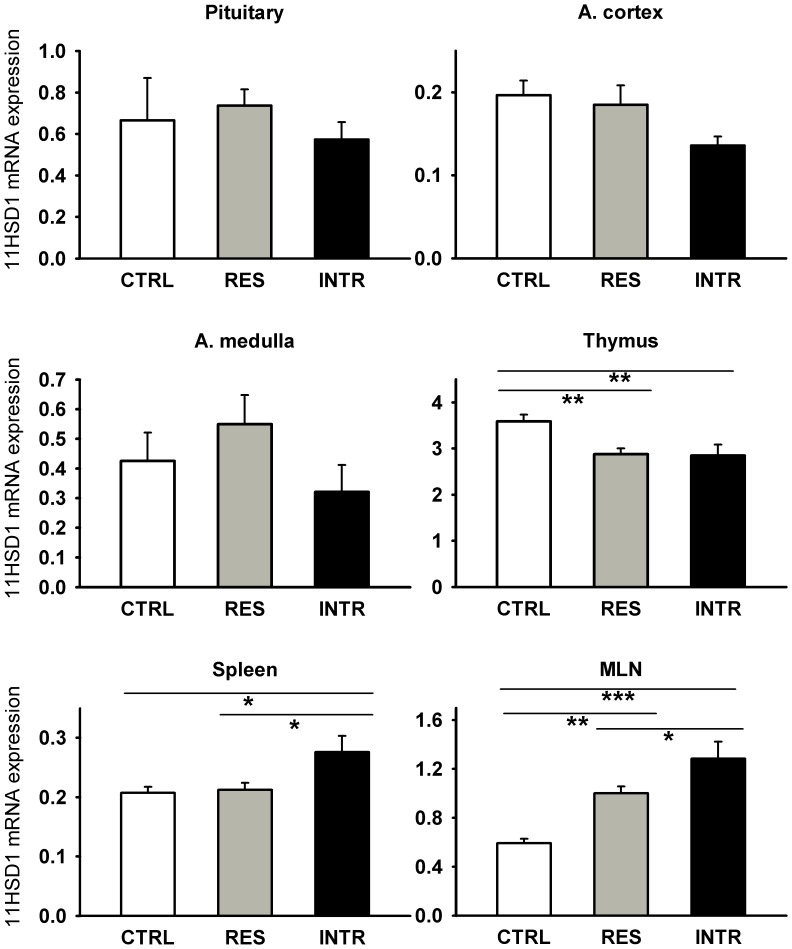
Effect of repeated social stress on expression of 11HSD1 in lymphoid organs, pituitary and adrenal cortex and medulla. CTRL, control rats; RES, resident rats; INTR, intruder rats; MLN, mesenteric lymphatic nodes. Data represent means ± SEM. *P<0.05, **P<0.01, ***P<0.001.

**Figure 6:**
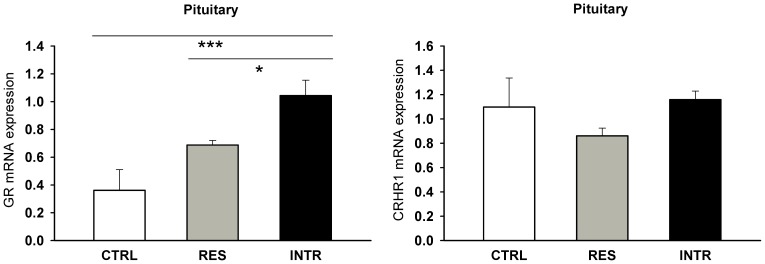
Expression of glucocorticoid (GR) and CRH receptors (CRHR1) in pituitary of control (CTRL), resident (RES) and intruder rats (INTR) after repeated social stress. All values are means ± SEM. *P<0.05, ***P<0.001.

Similar to PVN, 11HSD1 expression was neither changed in the pituitary nor in the adrenal gland (Fig. 5) that constitute the principle components of the HPA axis. However, the social stress provoked changes in the expression of GR in the pituitary (F_2,18_ = 5.17; P<0.001) and upregulated it more in intruders than in residents (Fig. 6). In contrast, the expression of CRHR1 was not changed, even if the stimulatory action of stress-induced CRH release is mediated primarily through binding to this receptor and CRH expression in PVN was significantly upregulated in both residents and intruders (Fig. 3).

Social stress has profound influence on immune responses [24], [25] and thus we measured also the expression of 11HSD1 in primary and secondary lymphoid organs of resident and intruder rats. As summarized in Fig. 5, the repeated social stress significantly modulated 11HSD1 in the lymphoid organs. 11HSD1 was upregulated in the spleen (F_2,17_ = 5.44; P<0.05) and MLN (F_2,17_ = 14.83; P<0.001) and this effect was more intensive in intruders than in resident rats. In the thymus, the effect was the opposite (F_2,17_ = 53.96; P<0.0001). The weights of the spleen, thymus, and adrenal glands in the controls were not different from the groups of residents and intruders (data not shown).

### Effect of Repeated Social Stress on Expression of Cytochrome CYP7B1

**Figure 7:**
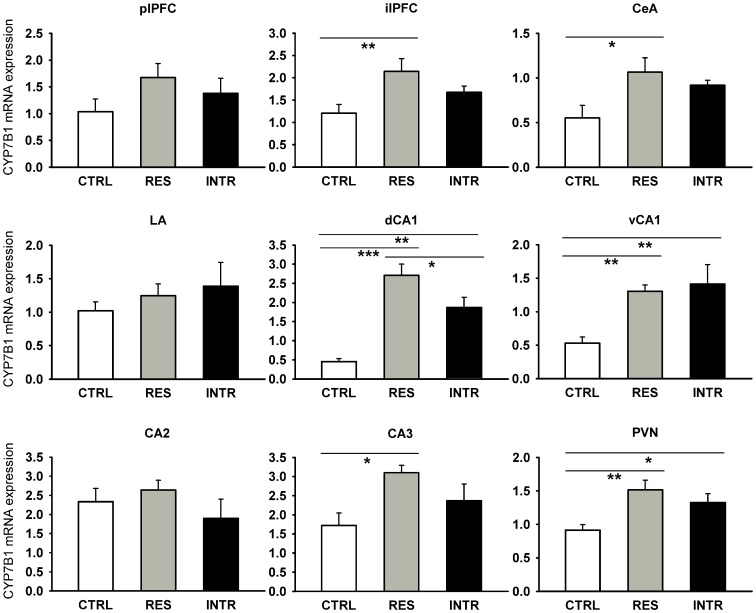
Effect of repeated social stress on expression of CYP7B1 in brain structures associated with the HPA axis. CTRL, control rats; RES, resident rats; INTR, intruder rats; plPFC, prelimbic prefrontal cortex; ilPFC, infralimbic prefrontal cortex; CeA, central amygdala; LA, lateral amygdala; dCA1 and vCA1, dorsal and ventral parts of CA1 hippocampus; CA2 and CA3, hippocampal CA2 and CA3 regions; PVN, paraventricular nucleus. All values are means ± SEM. *P<0.05, **P<0.01, ***P<0.001.

**Figure 8:**
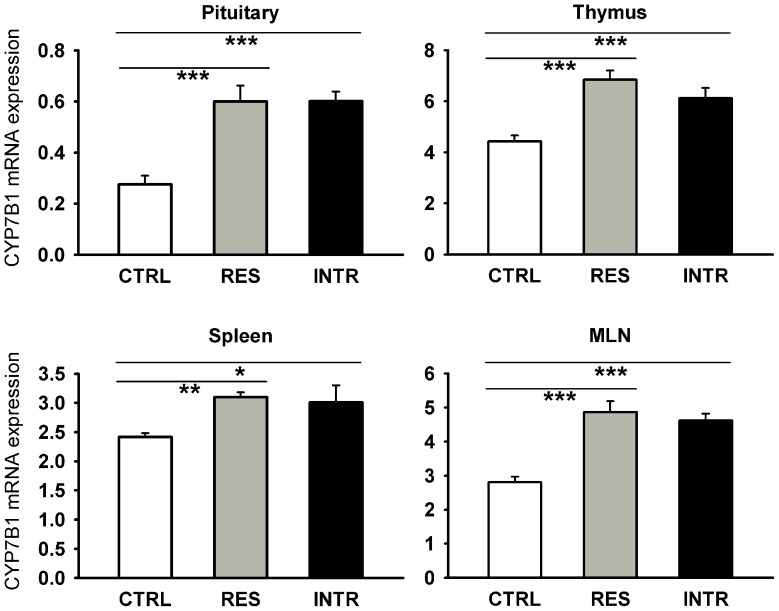
Effect of repeated social stress on expression of CYP7B1 in pituitary and lymphoid organs. CTRL, control rats; RES, resident rats; INTR, intruder rats. Data represent means ± SEM. *P<0.05, **P<0.01, ***P<0.001.

It has been postulated that 7-hydroxy-metabolites of C_21_ and C_19_ steroids, such as 7-hydroxy-dehydroepiandrosterone, can decrease local glucocorticoid levels by interaction with 11HSD1 [26]. In order to determine whether metabolism of 7-hydroxy-steroids may modulate 11HSD1 during stress, we examined the effect of social stress on CYP7B1, an enzyme, which catalyzes 7α-hydroxylation of steroids. The social stress used here lead to the upregulation of CYP7B1 in some brain structures, specifically, in the CeA (F_2,12_ = 4.51; P<0.05), ilPFC (F_2,12_ = 4.69; P<0.05), PVN (F_2,13_ = 5.67; P<0.05) and dCA1 (F_2,15_ = 19.31; P<0.0001), vCA1 (F_2,15_ = 7.58; P<0.001) and CA3 hippocampus (F_2,15_ = 4.93; P<0.05). As shown in Fig. 7, stress significantly increased CYP7B1 expression in the residents, and in the case of the PVN, dCA1, and vCA1 also in intruders. Moreover, stress stimulated the expression of CYP7B1 in the pituitary (F_2,17_ = 14.69; P<0.0001) and this effect was found in both groups of stressed animals (Fig. 8). These findings gave rise to the hypothesis that social stress might also modulate CYP7B1 expression in the lymphoid organs. The data summarized in Fig. 8 show that social stress caused a significant increase of CYP7B1 expression in both the primary (thymus: F_2,17_ = 11.67; P<0.0001) and secondary lymphoid organs (spleen: F_2,17_ = 4.54; P<0.05; MLN: F_2,17_ = 19.89; P<0.0001). Both residents and intruders exhibited a higher expression of CYP7B1 than the intact control rats.

## Discussion

In the present study, we report that chronic social stress upregulated the expression of enzymes that are able to modulate local concentration of glucocorticoids. Using the resident-intruder paradigm, we showed that manipulation with the social status of the animals for several consecutive days suppressed the social behavioral profile of the intruders and increased physiological stress markers (plasma corticosterone, CRH expression in PVN) in both intruders and residents, however, the intruders were stressed more than the residents. The results also demonstrate that short-term moderate repeated social stress did not increase the regulation of 11HSD1 mRNA in the principal components of the axis itself, even when 11HSD1 was previously detected in the PVN, anterior pituitary and adrenal glands [8]–[13]. In the adrenal glands, 11HSD1 expression was not influenced by the social stress, neither in the adrenal cortex nor in the medulla, where glucocorticoids are required for the normal functioning of chromaffin cells and their capacity to produce epinephrine [33]. However, the confrontation of resident and intruder increased the 11HSD1 mRNA in the secondary lymphoid organs and in the amygdala, prelimbic cortex and some regions of the hippocampus – the limbic structures that are activated by psychosocial stressors and are associated with the regulation of the HPA axis [5], [34]. Our findings are in agreement with the known role of the limbic structures in regulation of the HPA axis and with the sensitivity of these structures to glucocorticoids. Prelimbic cortex inhibits the HPA axis [4] and its activation reduces glucocorticoid secretion after stress [35] similar to corticosterone implants to this region [36]. Amygdala also appears to process glucocorticoid information, although there is functional differentiation among the individual amygdalar regions [5], [6]. Additionally, numerous studies indicate that hippocampus is involved in inhibiting the HPA axis response and expresses high levels of glucocorticoid receptors [37].

The stimulatory effect of stress in the vCA1 and CA2 regions of the hippocampus is consistent with a previous study of the effect of arthritic stress in rats on the whole hippocampus [19] but not with the finding of chronic psychosocial stress in tree shrews [20]. The reason for this discrepancy might reflect species-specific control of 11HSD1 or the type and duration of stress applied. As hippocampal cells reactivate inactive 11-dehydrocorticosterone to active corticosterone [38], we suggest that an increase in 11HSD1 expression after repeated social stress could modulate the local corticosterone concentration. The regional differences in the response of 11HSD1 mRNA to stress among CA subfields are difficult to reconcile with the hippocampal functions. Emerging evidence indicates that the dorsal hippocampus performs primarily cognitive functions, whereas the ventral part is connected to stress and emotion [39] and that corticosteroids have been shown to act as structural and functional modulators of limbic areas, including learning and memory [34], [40]. Comparison of the current findings with previous works indicates that corticosterone-sensitive neurons in limbic structures play a role in the feedback regulation of stress responses and thus the amplification of glucocorticoid signals due to upregulation of 11HSD1 might facilitate this feedback.

The action of glucocorticoids is predominantly mediated through intracellular lower-affinity glucocorticoid receptors (GR) that are activated by large amounts of glucocorticoids secreted during stress [34], [40]. We studied, therefore, not only the effect of chronic stress on 11HSD1 but also on GR. However, the expression of GR did not differ between the stressed and unstressed rats in all studied brain regions with the exception of the vCA1 hippocampus, even if the expression of 11HSD1 was upregulated in the prelimbic cortex, amygdala, and vCA1 and CA2 hippocampus. As GR are highly expressed in the hippocampus, prelimbic cortex and amygdala, which are critically involved in mediating stress-related behavior and modulating hippocampal functions [5], [40], the absence of changes in expression of GR together with upregulation of 11HSD1 indicates that these limbic structures undergo an adaptive corticosteroid-signaling reaction during repeated social stress based on 11HSD1 and not on GR. This reaction does not occur in the principal components of the HPA axis, such as the pituitary and adrenal gland, or in the intrahypothalamic regulatory nuclei such as the PVN, even if all of these structures express 11HSD1. In light of these facts, it can be hypothesized that the upregulation of 11HSD1 in amygdala, prelimbic cortex and some areas of hippocampus might intensify the glucocorticoid signal via activating GR due to conversion of plasma 11-dehydrocorticosterone to corticosterone and thus might subsequently attenuate the HPA axis via the activation of stress-inhibitory and damping of stress-excitatory regions of limbic structures [4].

Besides the brain, the social stress also modulated 11HSD1 expression in primary and secondary lymphoid organs. The stimulatory effect of stress on 11HSD1 in the spleen and MLN is in agreement with the suppression of immune responses as the most-reported consequence of stress [3]. Mucosal immunosuppression paralleled by epithelial barrier defects were found in murine social-stress-induced colitis [25] and we have recently shown an upregulation of 11HSD1 in the spleen and lymphatic nodes during colitis [15]. This amplification of 11HSD1 might be related to the action of proinflammatory mediators, since exposure to TNFα and IL-1β increases 11HSD1 [41], [42] and social stress upregulates plasma level and tissue gene expression of IL-1 cytokines [43]. Splenic and lymphatic node 11HSD1 upregulation and increased glucocorticoid regeneration might be part of the immunosuppresive effects of social stress, even if the reason for the discrepant observations between the secondary lymphoid organs and thymus are not clear.

How exactly social stress modulates 11HSD1 mRNA expression remains unknown. We can only speculate about a feasible mechanism based on the knowledge of regulation of 11HSD1 gene expression and neurohumoral and humoral factors secreted during stress. Traditionally, CRH, ACTH, catecholamines and glucocorticoids have been attributed to stress acclimation. However, studies investigating the effect of these humoral factors are limited and their results contradictory [19], [21], [50], [51]. Sequence analysis of 11HSD1 revealed several putative binding sites for various transcription factors, particularly CCAT/enhancer binding proteins (C/EBPs), AP1 (Fos/Jun), and NF-κB [44]–[47] and several studies have linked the regulation of 11HSD1 to these factors. Overexpression of AP1, C/EBPα and C/EBPβ potently increases 11HSD1 promoter activity, whereas overexpression of NF-κB rather inhibits this activity [44], [45]. Depending on cell types, several studies have linked TNFα to 11HSD1 upregulation via C/EBP-, AP1-, NF-κB-, or MAPK-signaling pathways [45], [46], [48] and OPN has been shown to activate NF-κB via degradation of NF-κB inhibitor IKβ [49]. In addition, recent data provide evidence for an indirect interaction of GR with 11HSD1 promoter via C/EBPβ transcription factor [52], [53].

Adding to the complexity of 11HSD1 regulation during social stress, we demonstrated here that the same stress protocol is able to enhance the expression of CYP7B1 both in brain and peripheral tissues. This cytochrome P450 catalyses the 7α-hydroxylation of steroids that subsequently interact with 11HSD1 and may direct the fine tuning of glucocorticoids [26]. An interesting hypothesis is that the upregulation of CYP7B1 by stress increases local concentration of 7α-derivatives and thus transformation of inactive 11-dehydrocorticosterone into active corticosterone may be modulated when 11HSD1 is faced with 7α-derivatives at the same time. This hypothesis is supported by several findings. First, a positive correlation was shown between increased CYP7B1 mRNA/CYP7B1 enzyme activity and the progression (severity) of murine arthritis [54]. Second, elevated IL-1β increased CYP7B1 activity [54]. Third, 7-OH metabolites of dehydroepiandrosterone counteracted glucocorticoid-induced apoptosis of murine splenocytes [55], and fourth, the directionality of CYP7B1 reaction depended crucially on the level of pyridine dinucleotide cosubstrates in endoplasmic reticulum [56], which can be modulated during psychosocial stress.

In summary, we provide evidence for the role of social stress in the regulation of the enzymes of local metabolism of glucocorticoids in specific brain structures and in lymphoid organs. The role of stress on expression of 11HSD1 and CYP7B should be kept in mind when studying the neuronal regulation of the stress reaction and the stress-associated changes in immune and inflammation responses.
